# Prevalence and risk factors of gastroesophageal reflux disease symptoms in Mazandaran, North of Iran: A Tabari cohort study

**DOI:** 10.22088/cjim.15.2.280

**Published:** 2024

**Authors:** Iradj Maleki, Samaneh Borhani, Mahmood Moosazadeh, Reza Alizadeh-Navaei

**Affiliations:** 1 Gut and Liver Research Center, Non-communicable Diseases Institute, Mazandaran University of Medical Sciences, Sari, Iran; 2 Gastrointestinal Cancer Research Center, Non-communicable Diseases Institute, Mazandaran University of Medical Sciences, Sari, Iran

**Keywords:** Gastro-esophageal reflux disease, prevalence, risk factor, epidemiology, cohort

## Abstract

**Background:**

Gastro-esophageal reflux disease (GERD) is a very common complaint. It is a major health concern and there is paucity of information about the epidemiology of the disease and its risk factors in Iran, especially Mazandaran province (North of Iran). This study aimed at investigating the prevalence of regurgitation and the factors associated with this condition in Tabari cohort study.

**Methods:**

This was a cross-sectional study that analyzed data from Tabari cohort study. Information including the presence and frequency of heartburn and regurgitation, demographic characteristics, socioeconomic status, occupational history, history of chronic illnesses, history of alcohol and cigarette consumption were recorded.

**Results:**

The prevalence of GERD symptoms were 27.6% (20.4% in men, and 32.4% in women, p=0.0001). The frequency of typical symptoms was significantly higher in women than that in men. The risk of developing GERD symptoms were 1.7 times higher in women (p=0.0001). The highest prevalence of GERD symptoms was found in urban areas (41.8%, p=0.0001), in people with low educational levels (48%, p=0.0001), and in participants with history of depression symptoms (36.2%, p=0.0001). The prevalence of GERD symptoms was significantly high in individuals with higher BMI (29.5%, p=0.006), greater waist to hip ratio (29.1%, p=0.0001, p=0.0001), and high waist circumference (31.7%, p=0.0001).

**Conclusion:**

This study showed gender, region of residence, educational level, and depression symptoms as the main risk factors for developing GERD symptoms.

Gastro-esophageal reflux disease (GERD) is a common illness that affect a majority of general population,The global prevalence of GERD has been 13.98% in recent systematic review (1). Evidence suggests that the prevalence of GERD has increased over the past two decades. If the process continues, it can cause a rapid increase in the incidence of serious complications, such as esophageal adenocarcinoma and causes high burden on healthcare systems (2). Nowadays, different diagnostic methods are used to diagnose the disease, but none are considered as the gold standard method (3). People with more severe symptoms or more frequent reflux, have lower health-related quality of life, work efficiency, and lower quality of sleep (4-6). The most dangerous complication of this disease is esophageal cancer (7, 8). Symptoms of the disease impair the quality of life even more than duodenal ulcers, untreated hypertension, mild congestive heart failure, angina or menopause (9). The disease also affects economic outcomes and decreases self-efficiency and increases work absences (10). The direct and indirect costs of this disease on healthcare systems are 111.4 $ per person annually (11).

A range of drugs are used in the treatment of GERD and acid suppressants are the main treatment (4). Using proton pump inhibitors (PPIs) are considered as the standard treatment for GERD (12). PPIs are the most potent acid suppressors and have shown efficacy to improve esophagitis and relieve symptoms, but about one-third of patients with GERD have persistent symptoms and poor response to standard dose PPI therapy (once daily) (11). To the best of our knowledge, there is a limited population-based epidemiological study on GERD and its related factors in Iran, so, this study was carried out to determine the prevalence of reflux disease symptoms and its related factors in people participating in Tabari population-based cohort study.

## Methods

**Study design:** In this study, part of the data collected during the Tabari cohort enrollment phase was used. Tabari cohort is part of a national cohort called Prospective Epidemiological Research Studies in Iran (PERSIAN) (13, 14). 

In the enrollment phase of Tabari cohort, 10255 people aged 35-70 years old enrolled that lived in urban (7012) and rural areas (3243) of Sari, Mazandaran province. Data including demographic characteristics, anthropometric indices, and blood pressure were recorded. Also, blood, urine, hair, and nail samples were collected (15). Age- and sex-matched controls were also selected from the Tabari cohort population. The standardized PERSIAN cohort questionnaire was administered which included demographic characteristics, socioeconomic status, occupational history, history of chronic illnesses, history of alcohol and cigarette consumption. The presence or absence of epigastric pain, heart burn and its frequency, the presence and frequency of regurgitation per year, any patients who presented by heartburn and/or regurgitation more than two times per week were considered as GERD symptoms. 

**Data analysis:** Data were analyzed in SPSS V18, applying t-test and chi-square test. Logistic regression was also used to report the odds ratio as raw and matched values to determine a more precise relationship between GERD and potential risk factors. In interpreting the results, factors with OR at least 1.5 were considered as risk factors (16). In all cases, p<0.05 was considered significant.

## Results

Typical symptoms of GERD, including epigastric pain and heartburn, return of the stomach content back up to the esophagus, and previous diagnosis of reflux were observed in 21.2%, 8.3%, and 6.4% of the participants, respectively. The frequency of GERD symptoms in men and women were 20.4% and 32.4%, respectively (P = 0.0001) (table 1).

The frequency of epigastric pain and heartburn was significantly higher in women than that in men (15.5% and 5.7%, P = 0.0001, respectively). Regurgitation was found to be significantly higher in women (5.8%) than men (2.5%) (P = 0.0001). Epigastric pain and heartburn in men were reported to occur daily (11.4%), several times a week (27%), several times a month (27.4%), and sometimes (34.2%). Reflux of stomach contents into the esophagus on a daily basis was reported by 58% of male participants. In women, daily episodes of epigastric pain and heartburn and regurgitation happened in 20.4% and 50.6%, respectively (table 2). 

The results of multivariate logistic regression analysis for the probability of developing GERD symptoms based on demographic characteristics are shown in table 3. The likelihood to develop GERD symptoms in women was 1.70 times more than that in men. People with history of depression symptoms were 1.71 times more likely to develop GERD symptoms than those without this condition (CI: 1.46-2.01). Also, participants with hypertension were 1.18 times more at risk of developing GERD symptoms than those with normal blood pressure. 

We found no significant differences between other variables such as diabetes, body mass index (BMI), and history of smoking, alcohol and hookah and the risk of developing GERD symptoms (table 3). 

**Table 1:** Demographic and anthropometric data in patients with GERD symptoms and normal controls

**Variable**		**Patient** **N (%)**	**Control** **N (%)**	**Total** **N (%)**	**P-value**
**Sex**	Male	846 (20.4)	3303 (79.6)	4149 (100)	0.0001
	Female	1980 (32.4)	4126 (67.6)	6106 (100)	
**Age categories**	35-44	723 (21.7)	2608 (78.3)	3331 (100)	0.0001
	45-54	938 (27.8)	2441 (72.2)	3379 (100)	
	55-70	1165 (32.9)	2380 (67.1)	3545 (100)	
**Marital status**	Single/divorced	254 (30.4)	581 (69.6)	835 (100)	0.053
	Married	2572 (27.3)	6848 (72.7)	9420 (100)	
**Residence**	Urban	1356 (41.8)	1887 (58.2)	3243 (100)	0.0001
	Rural/mountainous	1470 (21)	5542 (79)	7012 (100)	
**Socio-economic status**	Level 1 (minimum)	856 (41.7)	1195 (58.3)	2051 (100)	0.0001
	Level 2	614 (29.9)	1438 (70.1)	2052 (100)	
	Level 3	518 (25.3)	1532 (74.7)	2050 (100)	
	Level 4	444 (21.6)	1607 (78.4)	2051 (100)	
	Level 5(maximum)	394 (19.2)	1657 (80.8)	2051 (100)	
**Smoking**	No	2631 (28.2)	6695 (71.8)	9326 (100)	0.0001
	Yes	195 (21)	734 (79)	929 (100)	
**Alcohol**	No	2687 (28.4)	6762 (71.6)	949 (100)	0.0001
	Yes	139 (17.2)	667 (82.8)	806 (100)	
**Hookah**	No	2694 (27.9)	6978 (72.1)	9672 (100)	0.006
	Yes	132 (22.6)	451 (77.4)	583 (100)	
**Diabetes**	Present	518 (29.3)	1247 (70.7)	1765 (100)	0.064
	Not present	2308 (27.2)	6182 (72.8)	8490 (100)	
**Hypertension**	Present	769 (33.7)	1512 (66.3)	228 (100)	0.0001
	Not present	2057 (5.8)	5917 (74.2)	7974 (100)	
**History of depression symptoms**	Present	293 (36.2)	517 (63.8)	810 (100)	0.0001
	Not present	2533 (26.8)	6912 (73.2)	9445 (100)	
**BMI**	<25	669 (27.1)	1804 (72.9)	2473 (100)	0.006
	25-29	1142 (26.3)	3201 (73.7)	4343 (100)	
	≥30	1015 (29.5)	2424 (70.5)	3439 (100)	
**WHR**	≤0.9 in men or ≤0.85 in women	784 (24.2)	2461 (75.8)	3245 (100)	0.0001
	More than 0.9 in men or more than 0.85 in women	2042 (29.1)	4968 (70.9)	7010 (100)	
**Waist circumference**	Less than 102 cm in men or less than 88 cm in women	1234 (23.6)	3995 (76.4)	5229 (100)	0.0001
	≥ 102 cm in men or ≥88 cm in women	1592 (31.7)	3434 (68.3)	5026 (100)	
**Level of Education**	University/college	416 (17.5)	1958 (82.5)	2374 (100)	0.0001
	9-12	589 (20.3)	2307 (79.7)	2896 (100)	
	6-8	279 (24.8)	843 (75.2)	1121 (100)	
	1-5	807 (34.6)	1525(65.4)	2332 (100)	
	Illiterate	736 (48)	796 (52)	1532 (100)	

BMI= body mass index, WHR= waist–hip ratio

**Table 2:** Frequency of typical GERD symptoms by gender in general population

**Sex**	**Male** **N (%)**				**Female** **N (%)**				**P- value**
**Symptoms**	**sometimes**	**Several times a month**	**Several times a week**	**Almost daily**	**sometimes**	**Several times a month**	**Several times a week**	**Almost daily**	
Epigastric pain and heartburn	518 (34.2)	415 (27.4)	409 (27)	172 (11.4)	864 (26.6)	751 (23.6)	938 (29.5)	650 (20.4)	0.0001
Reflux of gastric contents to the esophagus	54 (3.9)	203 (14.5)	330 (23.6)	810 (58)	166 (7.8)	425 (19.9)	463 (21.7)	1078 (50.6)	0.0001

**Table 3:** Results of multivariate logistic analysis for the probability of developing GERD symptoms based on demographic and basic characteristics

**Variable**		**Probability of developing GERD** **(Odds Ratio)**	**CI 95%**	**P-value**
**Sex**	Male	-	-	-
	Female	1.70	1.50-1.92	0.0001
**Age categories**	35-44	-	-	-
	45-54	1.34	1.29-1.65	0.0001
	55-70	1.46	1.29-1.65	0.0001
**Marital status**	Single/divorced	-	-	-
	Married	1.23	1.4-1.45	0.014
**Occupancy**	Rural	-	-	-
	Urban	2.37	2.10-2.66	0.0001
**Socio-economic status**	Level 1 (minimum)	-	-	-
	Level 2	0.81	0.71-0.93	0.004
	Level 3	0.82	0.70-0.95	0.012
	Level 4	0.74	0.63-0.88	0.001
	Level 5(maximum)	0.68	0.57-0.81	0.0001
**Level of education**	University/college	-	-	-
	9-12	1.03	0.88-1.19	0.690
	6-8	1.20	0.99-1.45	0.053
	1-5	1.52	1.28-1.87	0.0001
	Illiterate	2.04	1.65-2.51	0.0001
**History of diabetes**		0.90	0.80-1.02	0.116
**History of hypertension**		1.18	1.05-1.32	0.003
**History of Depression symptoms**		1.71	1.46-2.01	0.0001
**History of smoking**		1.02	0.85-1.23	0.799
**History of drinking Alcohol**		1.03	0.83-1.27	0.782
**History of smoking hookah**		1.25	1.01-1.55	0.038
**BMI**	<25	-	-	-
	25-29	0.97	0.85-1.10	0.663
	≥30	0.97	0.82-1.14	0.743
**WHR**	≤0.9 in men and ≤0.85 in women	-	-	-
	More than 0.9 in men and more than 0.85 in women	1.03	0.92-1.16	0.558
**Waist circumference**	Less than 102 cm in men and less than 88 cm in women	-	-	-
	≥ 102 cm in men and ≥88 cm in women	1.17	1.01-1.35	0.031

BMI= body mass index, WHR= waist–hip ratio

## Discussion

In the present study, the prevalence of GERD symptoms was estimated to be 27.6% in people living in the North of Iran which is much lower compared to a similar study in southwest of Iran (58.5%) (17). Both studies indicated higher prevalence of GERD symptoms than other parts of the world (6% in France and 3.8% in China) (18, 19). However, the results of the current study are similar to the findings of a research in Saudi Arabia in which the prevalence of GERD was 28.7% (20). A systematic review showed that the prevalence of heartburn symptoms in US, Finland, and China was 7.8%, 15%, and 3.1%, respectively (21). In Greece, the prevalence of GERD symptoms was reported to be 52% (22) which is much higher than the rate observed in this study. The reasons for differences in prevalence of GERD in different studies could be due to sample size, methodology (cross-sectional or population-based), definition of GERD according to symptoms or signs, and dietary habits in different geographical areas.

The prevalence of GERD symptoms in men (20.4%) and women (32.4%), in our population, indicates an association between gender and GERD. This is in contrast with the results of Spantideas et al. that found no significant difference in the prevalence of GERD according to sex (22), or previous studies from Iran that reported higher prevalence of GERD in men (23, 24). We observed the highest prevalence of GERD in individuals aged 55 to 70 years old (32.9%) that was even higher in older people (greater effect size). These findings are in line with the results of other studies in Iranian (17) and Greek populations (22).

In the present study, history of depression symptoms, living in city and low levels of education were found as effective risk factors causing GERD symptoms (OR> 1.5). Saberi-Firoozi et al., also reported similar results (25). The significant correlation between GERD and depression could be due to the high prevalence of functional heartburn and esophageal hypersensitivity, which are other subgroups of reflux disease in Rome IV classification, that is directly correlated with increased prevalence of anxiety and depression (26). In this study, however, we were not able to prove this because the diagnosis was made only on the basis of typical symptoms and paraclinical procedures were not performed. The high prevalence of GERD in urban areas compared to rural areas may be related to psychosocial factors (27)

There are inconsistent data about the role of smoking in GERD. In the US and Sweden, cigarette smoking was found as an independent factor for developing reflux disease (28-30). But some studies showed no association between smoking and GERD, some studies reported no significant correlation between alcohol consumption and GERD (28, 31), while some substantiated the role of alcohol consumption as an independent factor in the development of GERD (17, 29, 32). In current study, the likelihood to develop GERD was slightly higher in people without history of hookah smoking (OR = 1.25). There was no relation between anthropometric indices (BMI and waist–hip ratio (WHR), OR =1) and the risk of developing the disease, and only WHR increased somewhat the risk of developing the disease (17%, effect size < 1.5). This result is consistent with previous studies in Iran (17, 25).

Evidence suggests no significant association between diabetes mellitus and GERD (32) and our study corroborates these results, but the probability of developing the disease was higher in our patients with hypertension (OR = 1.18, effect size< 1.5). Khodamaradi et al. also observed that patients with hypertension were 1.16 times more likely to develop GERD compared to non-hypertensive individuals (17).

The current study has strengths as well as weaknesses. The relatively large sample size makes our results more reliable. On the other hand, we performed a cross-sectional study in enrollment phase of a cohort study which was the weakness of this study.

GERD symptoms have negative impacts on individuals that interfere with daily activities and impaired quality of life. This population-based study with large sample size showed a high prevalence of GERD symptom in North of Iran and it was higher than western countries and countries of East Asia. Overall, this study showed that female gender, low level of socio-economic status, living in city, low levels of education, and history of depression symptoms could affect the development of GERD symptoms. Additionally, older age, waist circumference, history of hypertension and hookah smoking were the week risk factors for developing these symptoms, but, history of diabetes, cigarette smoking, drinking Alcohol, BMI and WHR were not significant relation with GERD symptoms.
